# Supplementary figures and images for: A Novel Peptide that Disrupts the Lck-IP3R Protein-Protein Interaction Induces Widespread Cell Death in Leukemia and Lymphoma
Source: Arch Microbiol Immunol. Author manuscript; Available in PMC 2023 Oct 12. (PMC10569261; doi:10.26502/ami.936500114)

Fig 1D

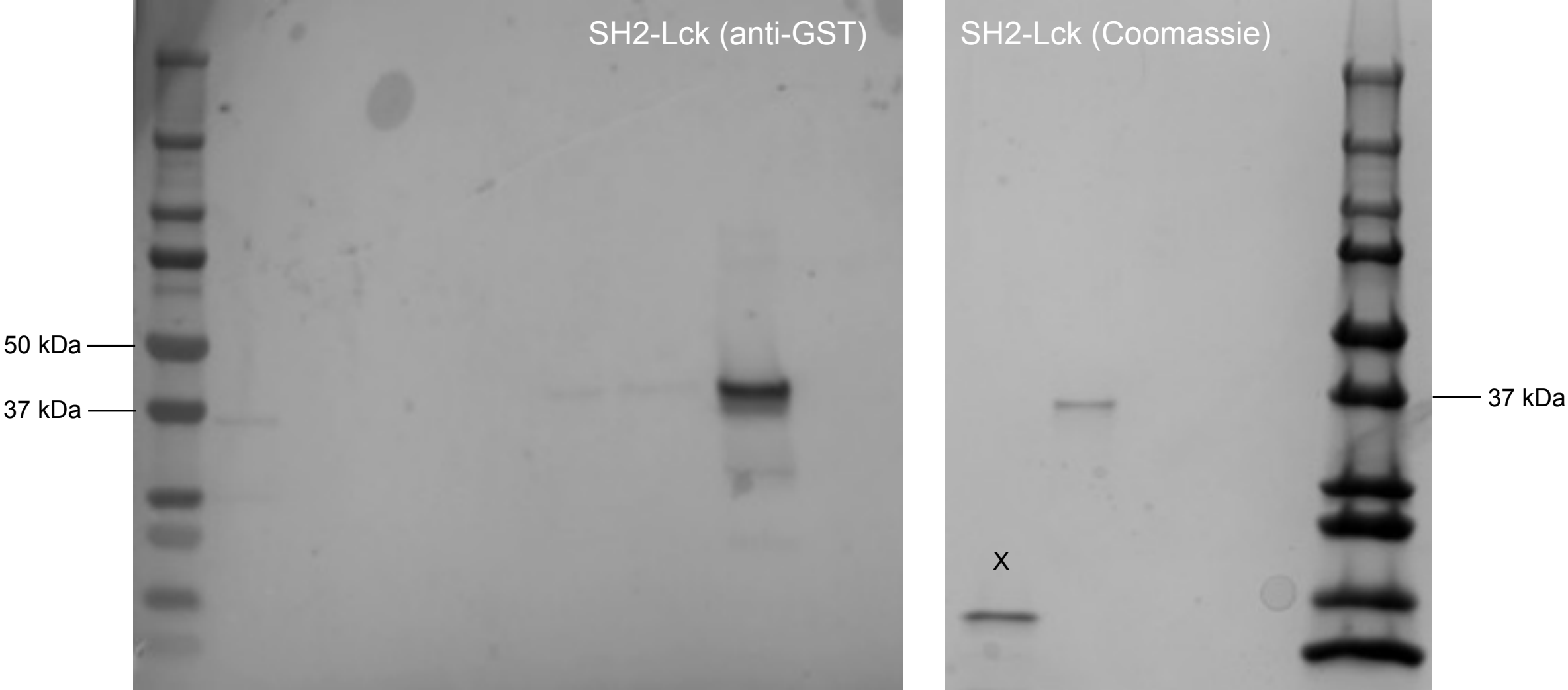

Fig 2A

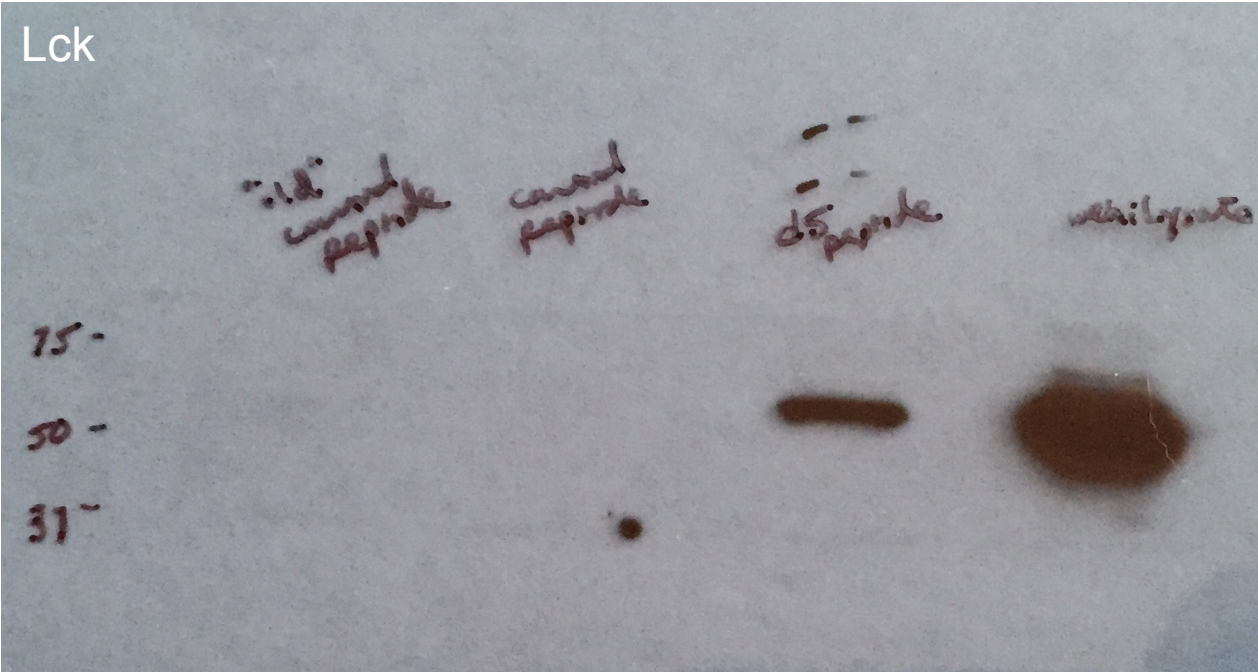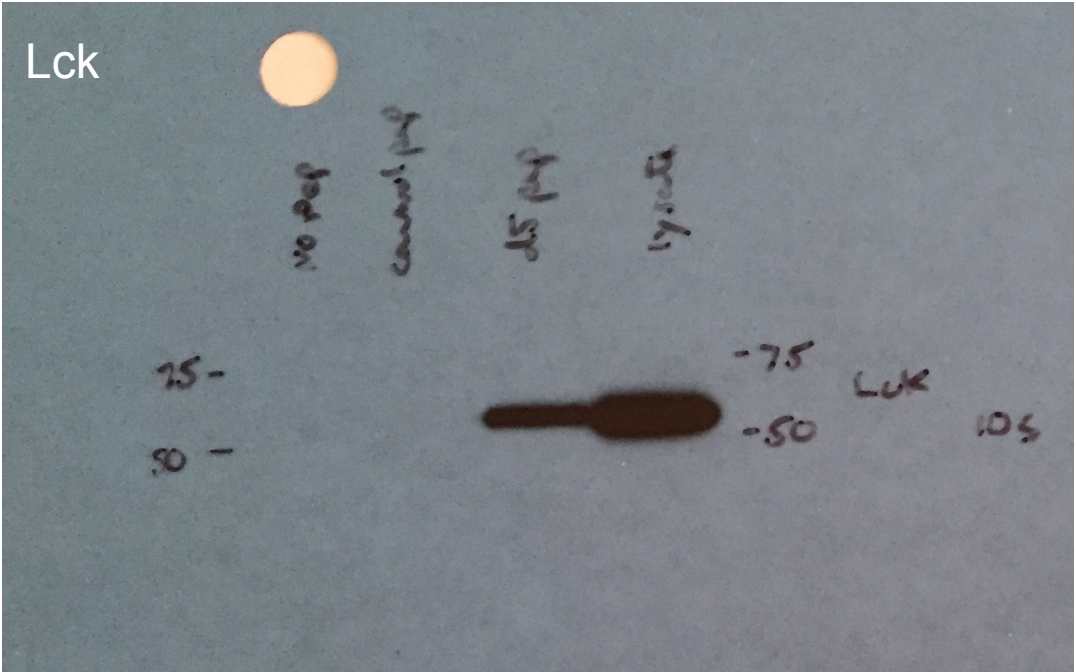

Fig 2B

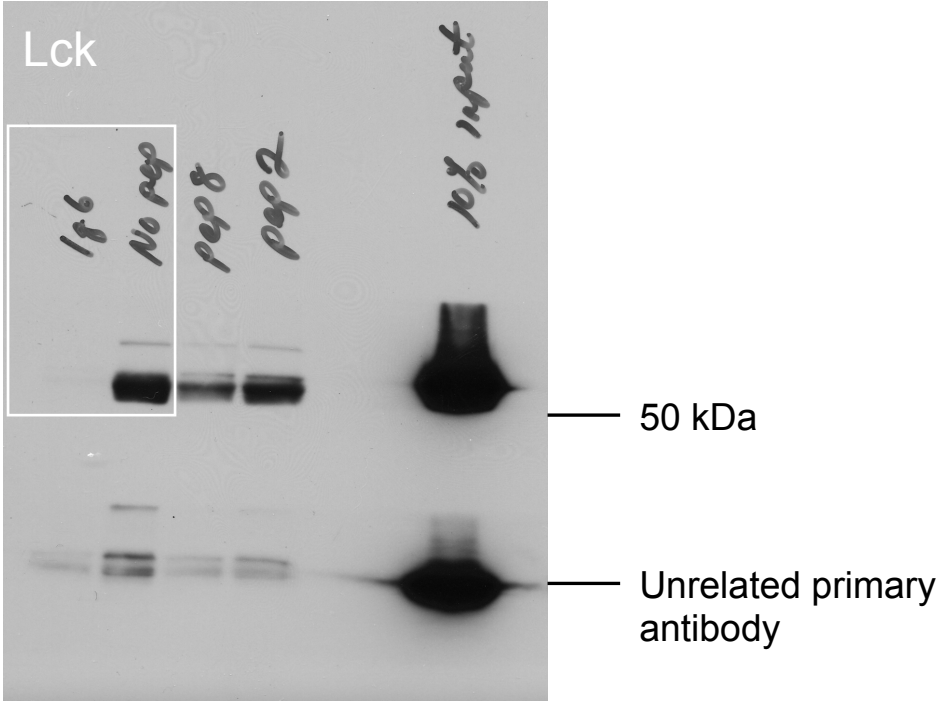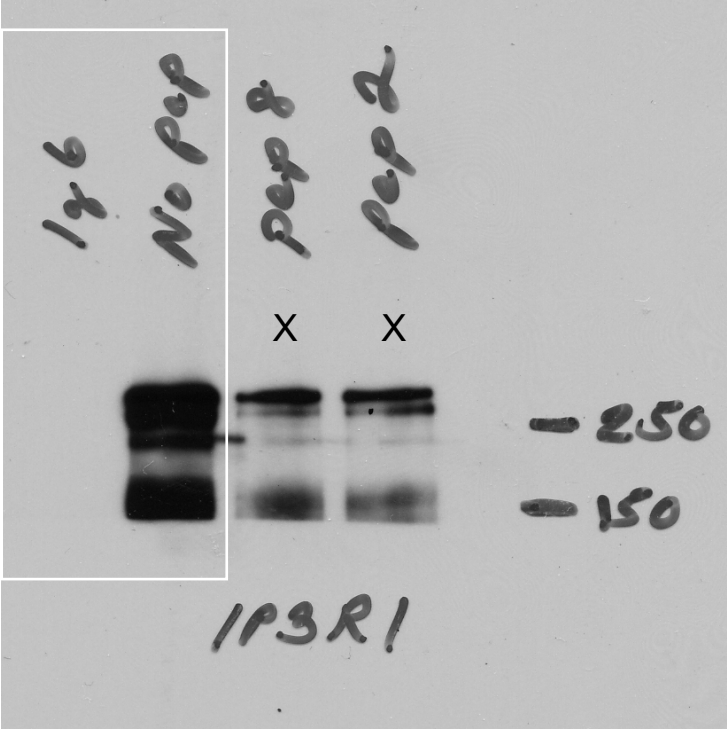

Fig 2C

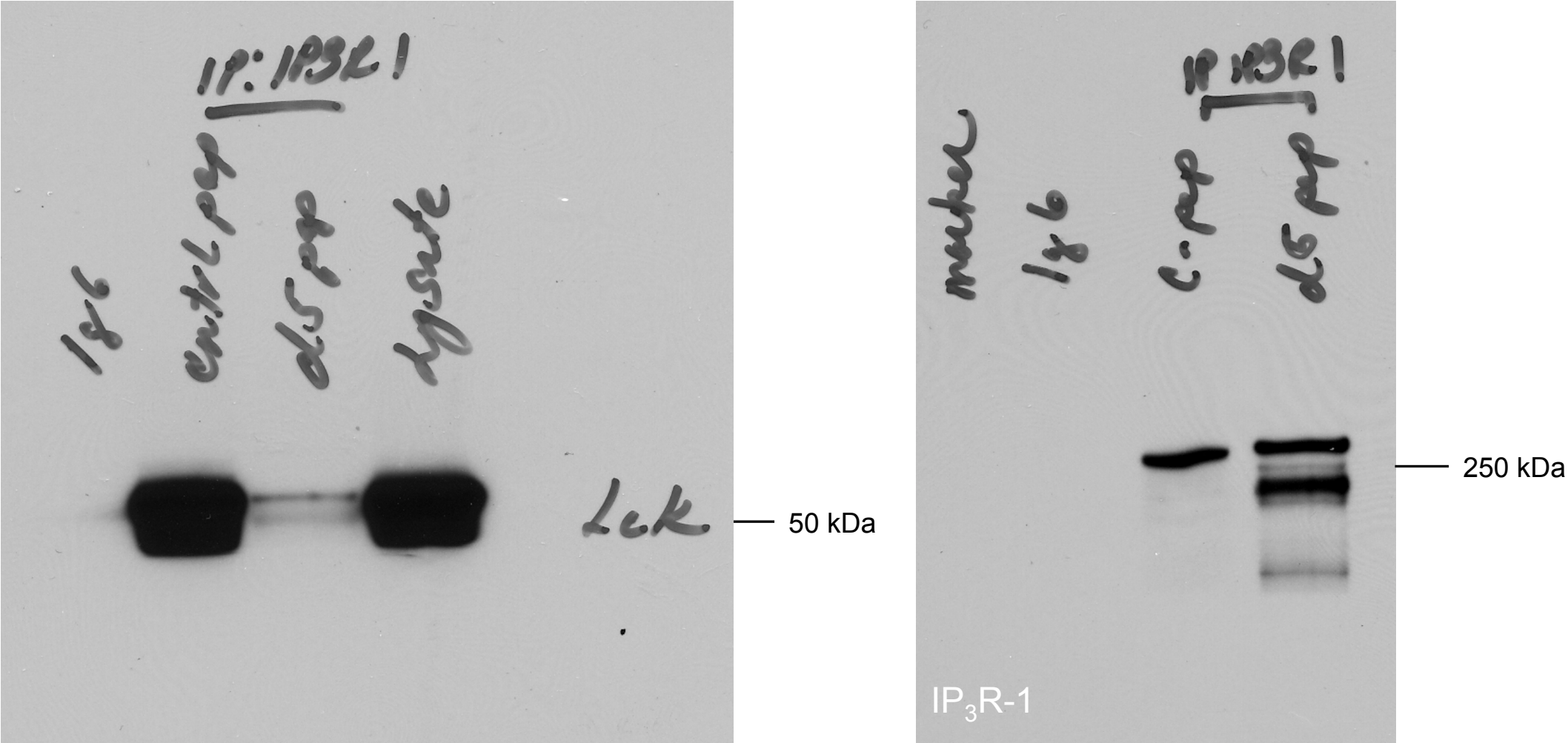

Fig 3B

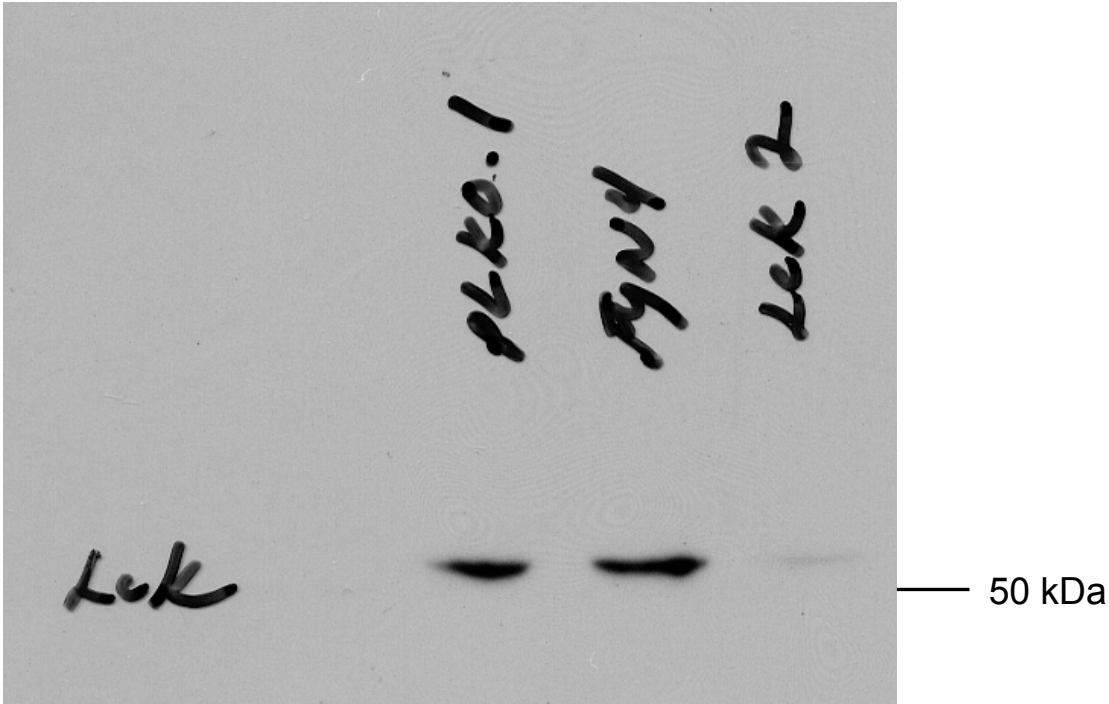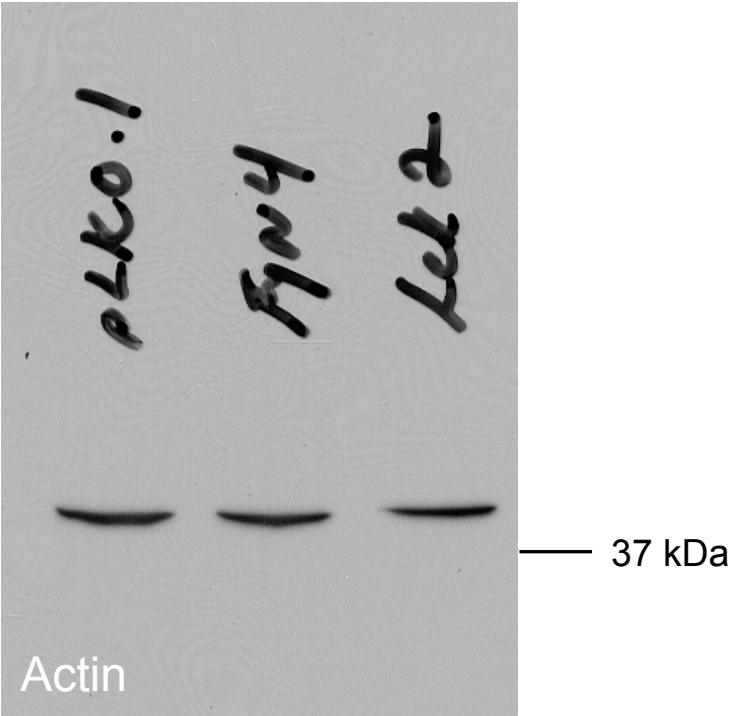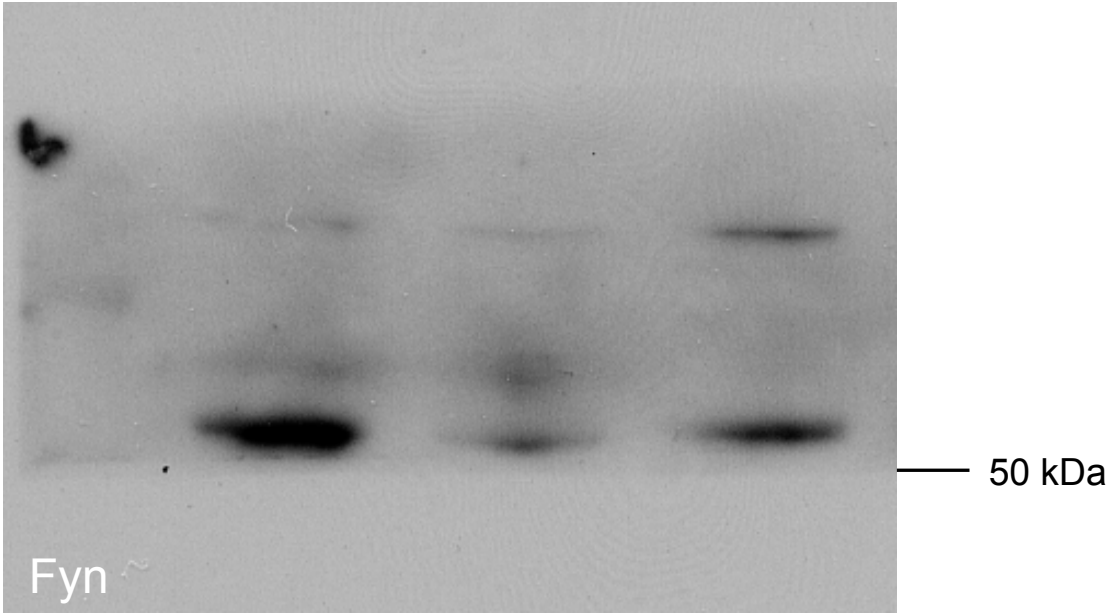

Fig 3E

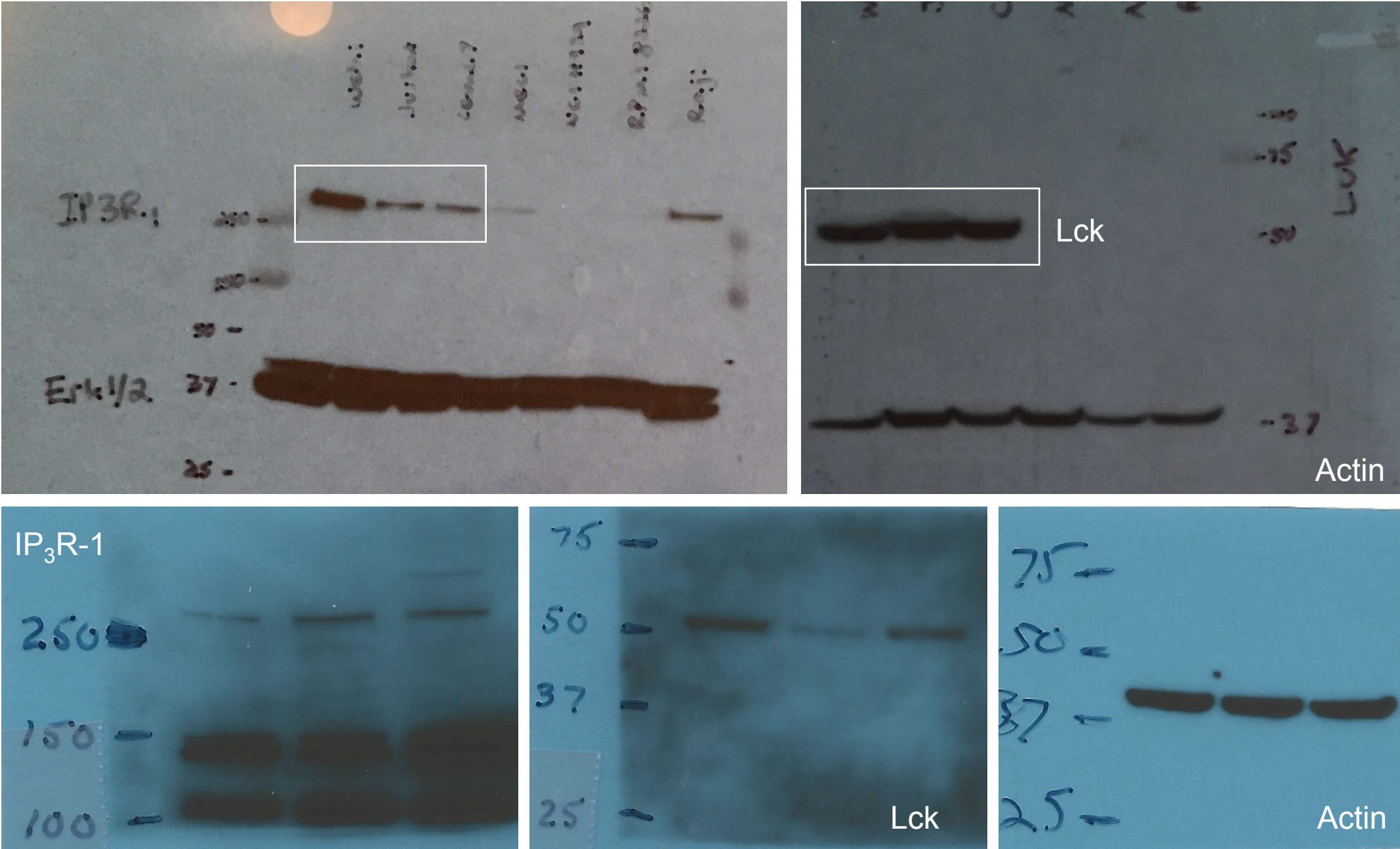

Fig 4A

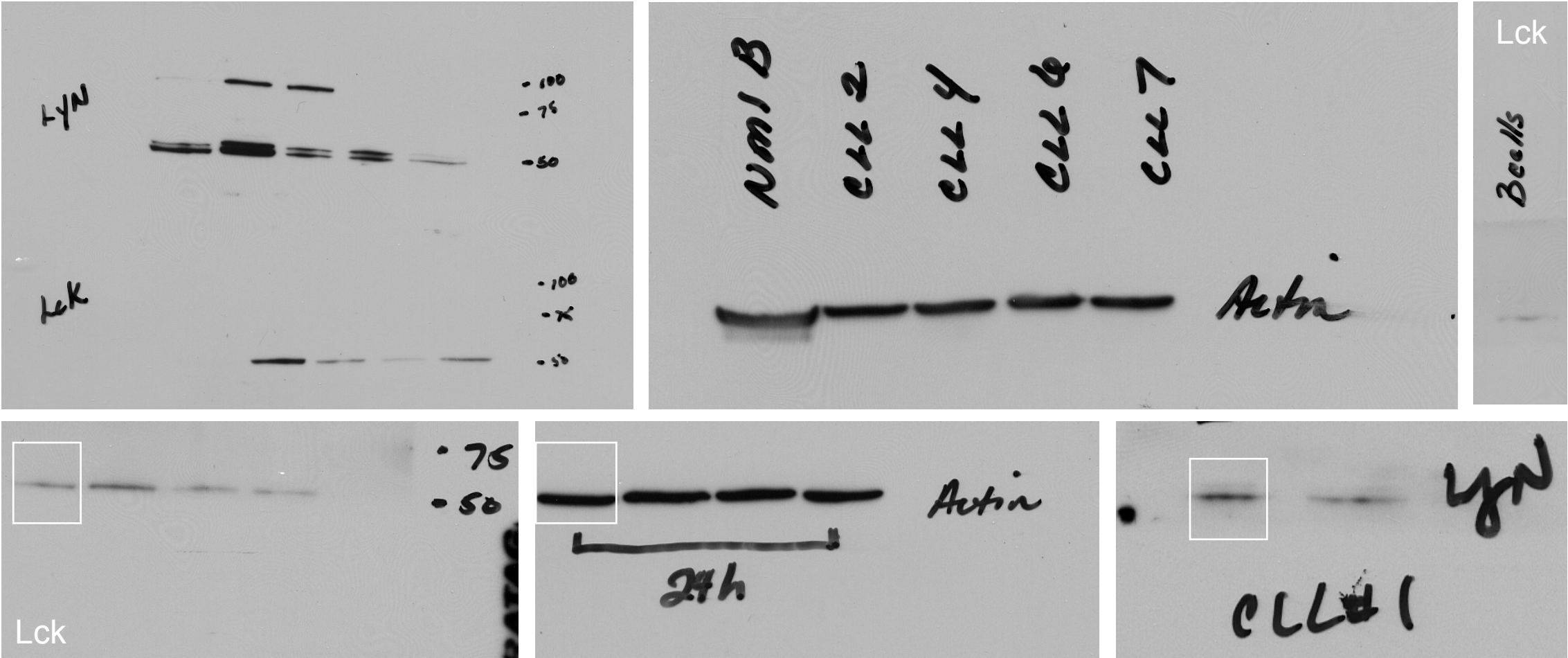

Fig 4B

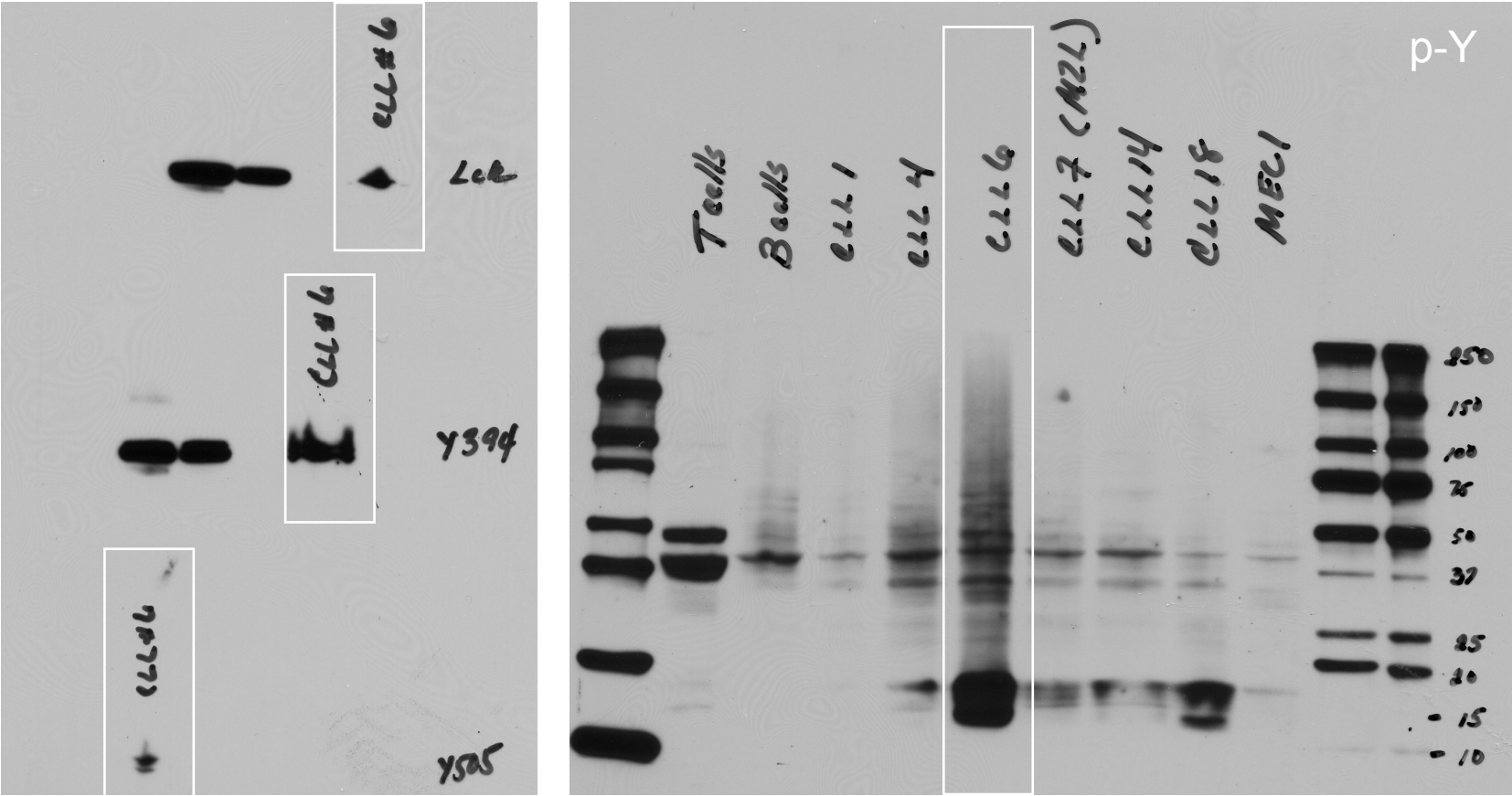

Fig 4G

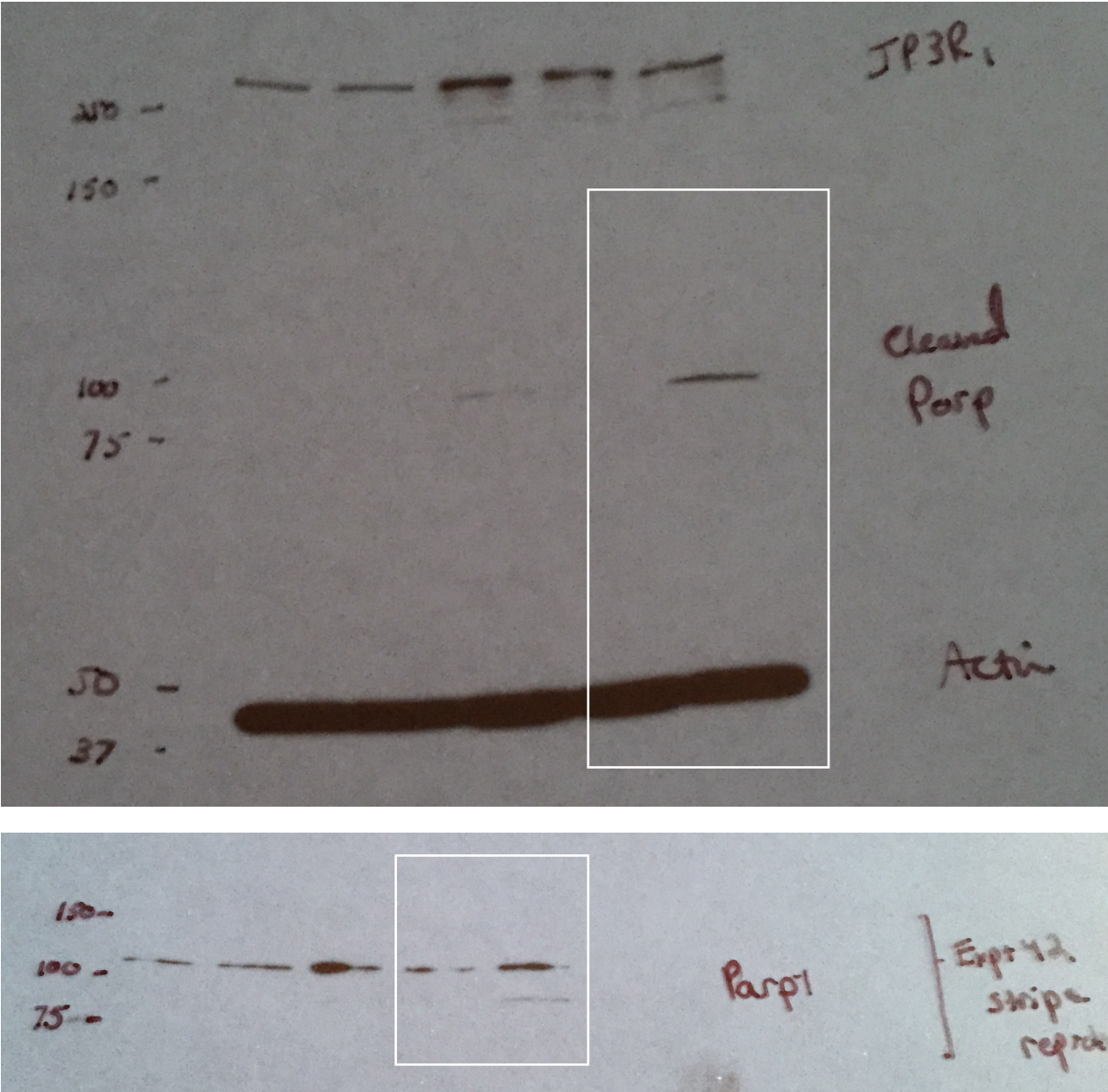

Fig S1

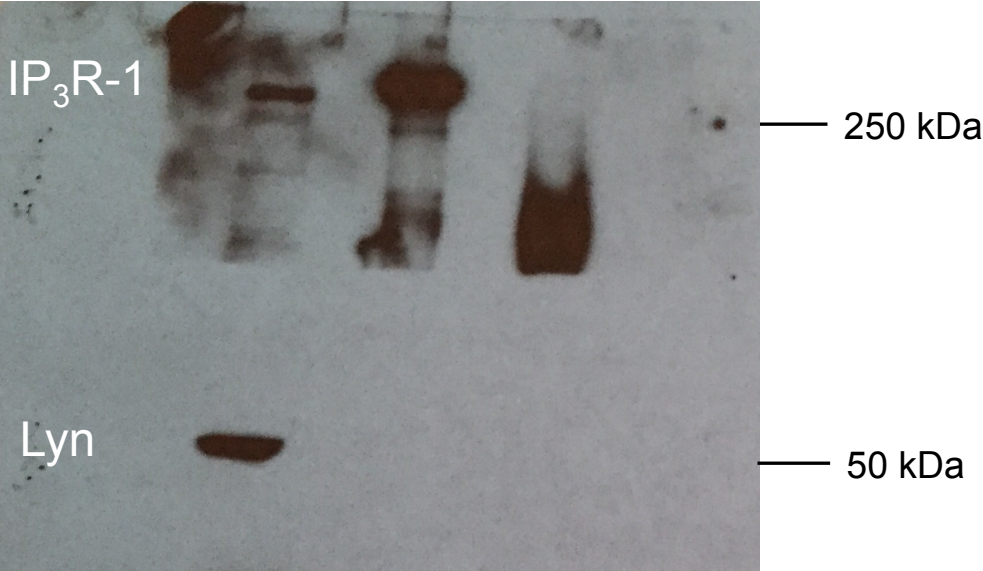

Supplement: Supplementary [file NIHMS1930806-supplement-Supplementary.zip › Supplimentary-files/8cf6349d5e12cd7d87a1c86b.pdf]

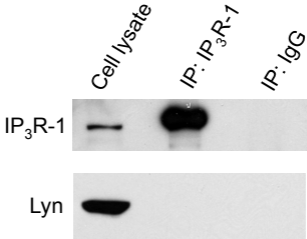

Supplement: Supplementary [file NIHMS1930806-supplement-Supplementary.zip › Supplimentary-files/Figure S1 (1).pdf]

A

TAT-D5SD

RKKRRQRRRGKKRMDLVLELKNNASKLLAI

TAT-ctrl

RKKRRQRRRGGNLNHSDQFAENLSHICGGHG

B

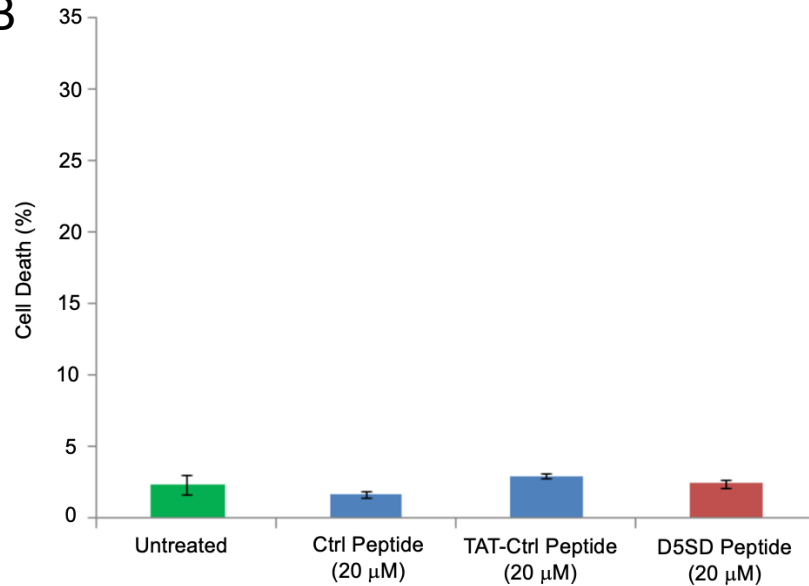

Supplement: Supplementary [file NIHMS1930806-supplement-Supplementary.zip › Supplimentary-files/Figure S2.pdf]

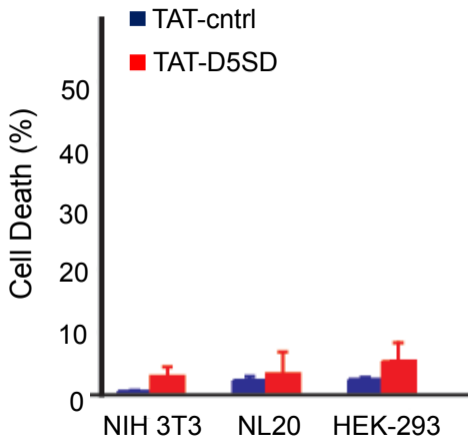

Supplement: Supplementary [file NIHMS1930806-supplement-Supplementary.zip › Supplimentary-files/Figure S3.pdf]

RNA Seq of primary CLL (N=68)

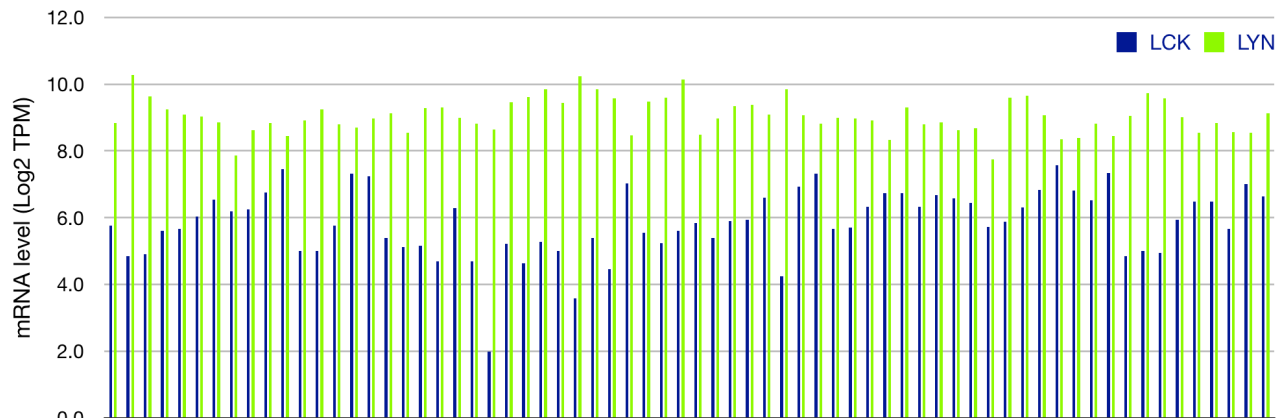

RNA Seq of primary B-cell lymphoma (N=103)

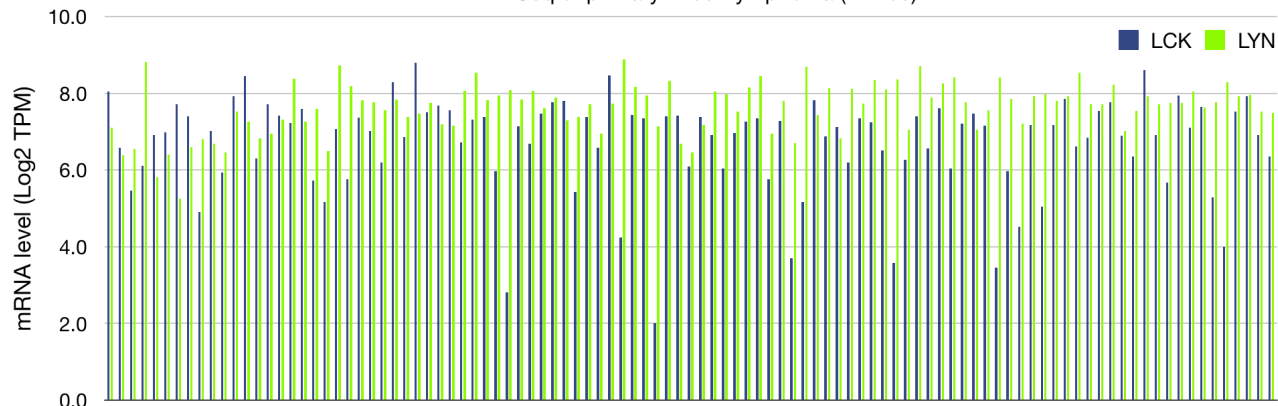

Supplement: Supplementary [file NIHMS1930806-supplement-Supplementary.zip › Supplimentary-files/Figure S4.pdf]

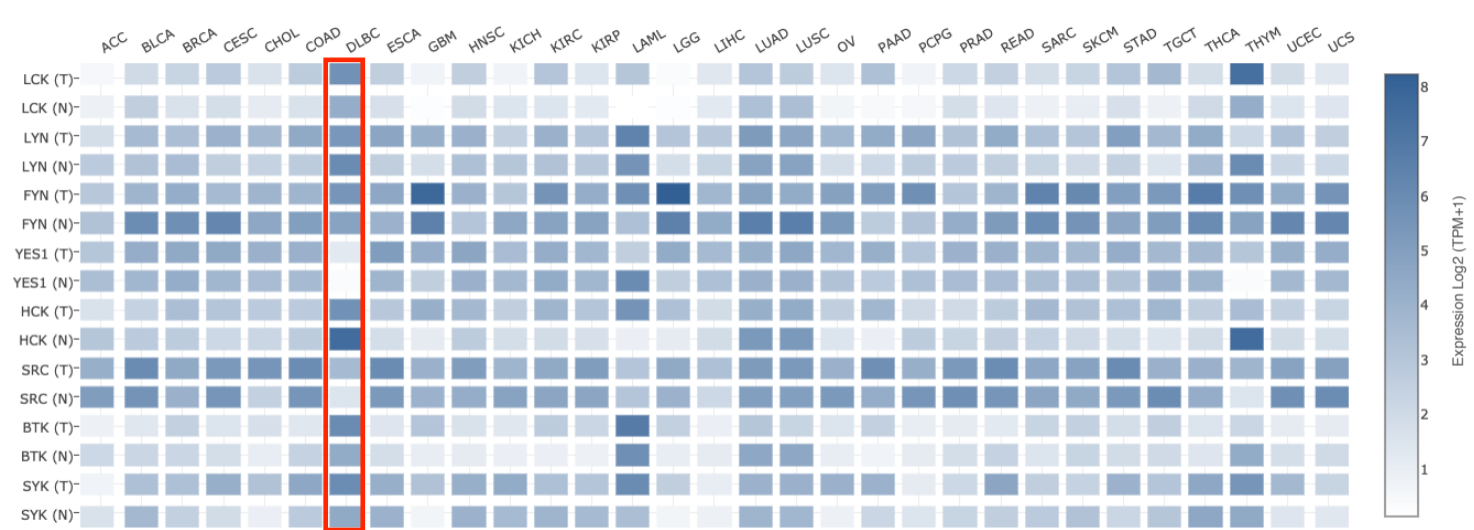

Supplement: Supplementary [file NIHMS1930806-supplement-Supplementary.zip › Supplimentary-files/Figure S5.pdf]

A

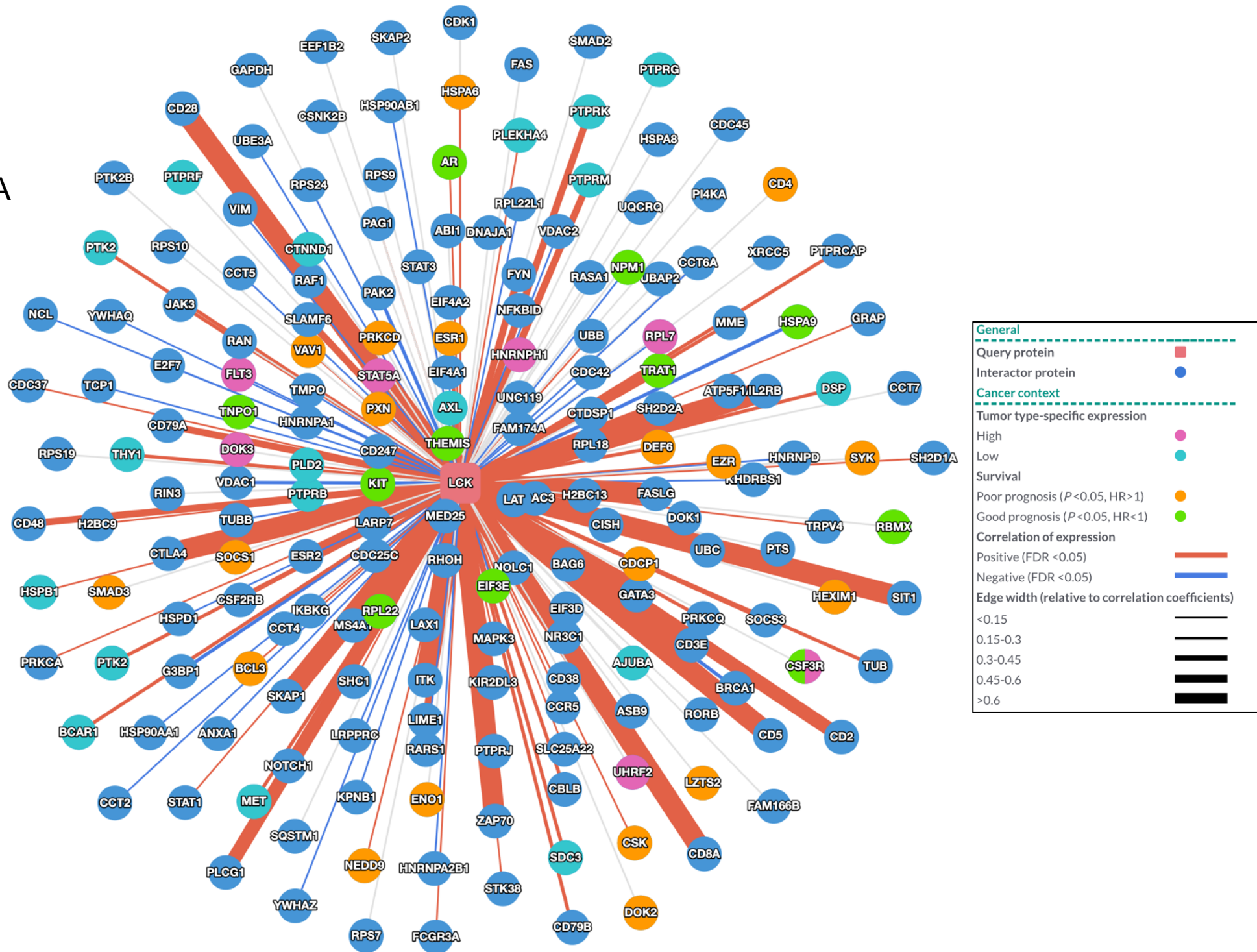

B

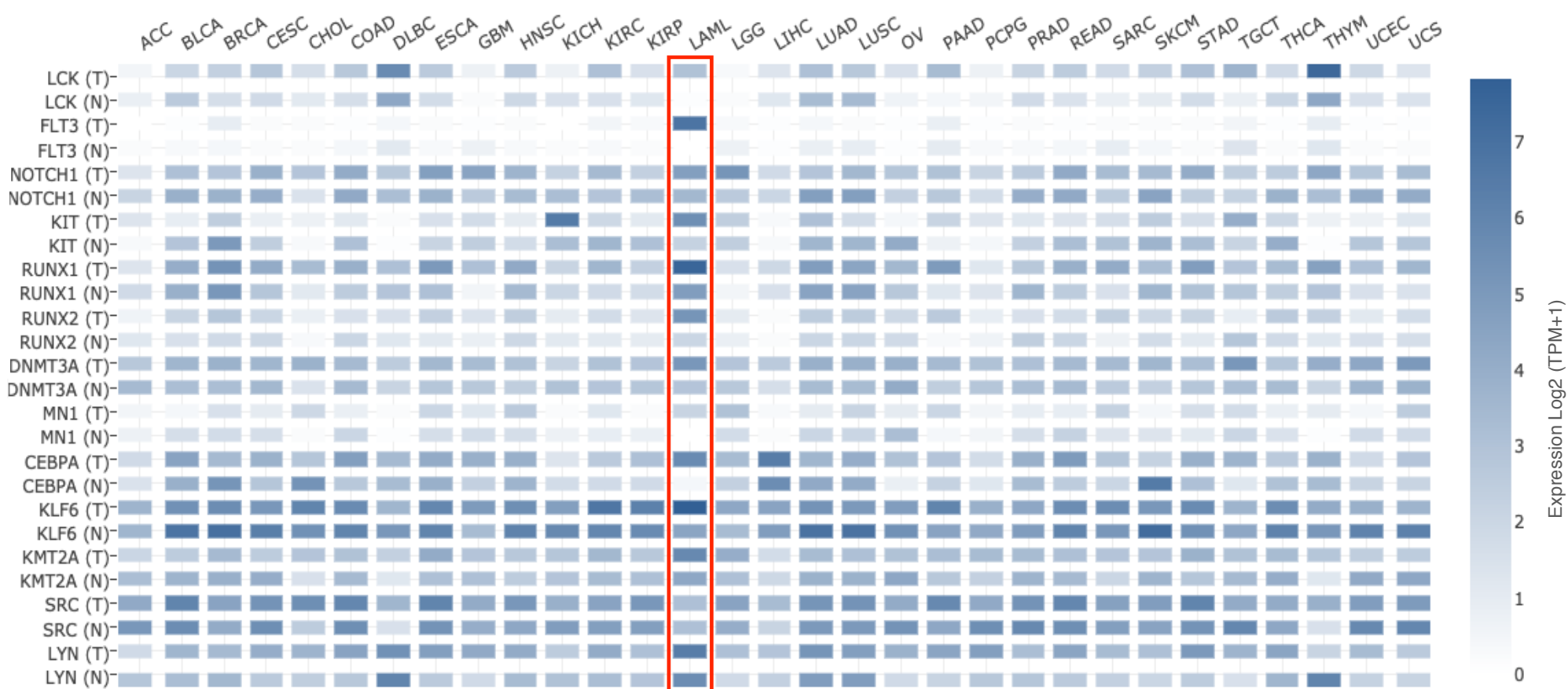

Supplement: Supplementary [file NIHMS1930806-supplement-Supplementary.zip › Supplimentary-files/Figure S6.pdf]
